# Bis([μ-bis­(diphenyl­arsino)methane-1:2κ^2^ *As*:*As*’]nona­carbonyl-1κ^3^ *C*,2κ^3^ *C*,3κ^3^ *C*-{tris­[4-(methyl­sulfan­yl)phen­yl]arsine-3κ*As*}-*triangulo*-triruthenium(0)) dichloro­methane monosolvate

**DOI:** 10.1107/S1600536810032812

**Published:** 2010-08-21

**Authors:** Omar bin Shawkataly, Imthyaz Ahmed Khan, Siti Syaida Sirat, Chin Sing Yeap, Hoong-Kun Fun

**Affiliations:** a Chemical Sciences Programme, School of Distance Education, Universiti Sains Malaysia, 11800 USM, Penang, Malaysia; b X-ray Crystallography Unit, School of Physics, Universiti Sains Malaysia, 11800 USM, Penang, Malaysia

## Abstract

The asymmetric unit of the title *triangulo*-triruthenium compound, 2[Ru_3_(C_25_H_22_As_2_)(C_21_H_21_AsS_3_)(CO)_9_]·CH_2_Cl_2_, consists of one *triangulo*-triruthenium complex mol­ecule and one half of a dichloro­methane mol­ecule which lies across a crystallographic inversion center, leading to the disorder of this mol­ecule over two positions of equal occupancy. The bis­(diphenyl­arsino)methane ligand bridges an Ru—Ru bond and the monodentate arsine ligand bonds to the third Ru atom. All arsine ligands are equatorial with respect to the Ru_3_ triangle. Each Ru atom carries one equatorial and two axial terminal carbonyl ligands. The three methyl­sulfanyl-substituted benzene rings make dihedral angles of 70.02 (8), 82.85 (9) and 89.49 (8)° with each other. The dihedral angles between the two phenyl rings are 78.25 (9) and 86.59 (9)° for the two diphenyl­arsino groups. In the crystal, weak inter­molecular C—H⋯π inter­actions are observed.

## Related literature

For general background to *triangulo*-triruthenium derivatives, see: Bruce *et al.* (1985 ▶); Bruce, Liddell, Hughes *et al.* (1988 ▶); Bruce, Liddell, Shawkataly *et al.* (1988 ▶). For related structures, see: Shawkataly *et al.* (1998 ▶, 2004 ▶); Shawkataly, Khan, Sirat *et al.* (2010*a* ▶,*b* ▶); Shawkataly, Khan, Yeap & Fun (2010*a* ▶,*b* ▶). For the synthesis of bis­(diphenyl­arsino)methane, see: Bruce *et al.* (1983 ▶). For the stability of the temperature controller used in the data collection, see: Cosier & Glazer (1986 ▶).
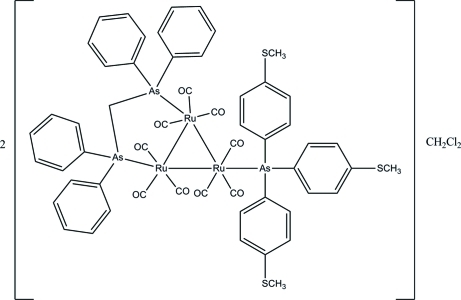


## Experimental

### 

#### Crystal data

2[Ru_3_(C_25_H_22_As_2_)(C_21_H_21_AsS_3_)(CO)_9_]·CH_2_Cl_2_
*M* *_r_* = 3029.01
Triclinic, 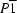

*a* = 10.8807 (1) Å
*b* = 12.7494 (2) Å
*c* = 20.9320 (3) Å
α = 94.512 (1)°
β = 98.721 (1)°
γ = 102.855 (1)°
*V* = 2779.13 (6) Å^3^
*Z* = 1
Mo *K*α radiation
μ = 2.79 mm^−1^
*T* = 100 K
0.56 × 0.29 × 0.20 mm

#### Data collection

Bruker SMART APEXII CCD area-detector diffractometer
Absorption correction: multi-scan (*SADABS*; Bruker, 2009 ▶) *T*_min_ = 0.306, *T*_max_ = 0.605
117127 measured reflections
20038 independent reflections
17668 reflections with *I* > 2σ(*I*)
*R*_int_ = 0.027

#### Refinement

*R*[*F* ^2^ > 2σ(*F* ^2^)] = 0.023
*wR*(*F* ^2^) = 0.059
*S* = 1.01
20038 reflections
679 parameters
H-atom parameters constrained
Δρ_max_ = 1.53 e Å^−3^
Δρ_min_ = −1.28 e Å^−3^

### 

Data collection: *APEX2* (Bruker, 2009 ▶); cell refinement: *SAINT* (Bruker, 2009 ▶); data reduction: *SAINT*; program(s) used to solve structure: *SHELXTL* (Sheldrick, 2008 ▶); program(s) used to refine structure: *SHELXTL*; molecular graphics: *SHELXTL*; software used to prepare material for publication: *SHELXTL* and *PLATON* (Spek, 2009 ▶).

## Supplementary Material

Crystal structure: contains datablocks global, I. DOI: 10.1107/S1600536810032812/is2586sup1.cif

Structure factors: contains datablocks I. DOI: 10.1107/S1600536810032812/is2586Isup2.hkl

Additional supplementary materials:  crystallographic information; 3D view; checkCIF report

## Figures and Tables

**Table 1:** Hydrogen-bond geometry (Å, °) *Cg*1, *Cg*2, *Cg*3, *Cg*4 and *Cg*5 are the centroids of the C26–C31, C32–C37, C1–C6, C14–C19 and C38–C43 benzene rings, respectively.

*D*—H⋯*A*	*D*—H	H⋯*A*	*D*⋯*A*	*D*—H⋯*A*
C3—H3*A*⋯*Cg*1^i^	0.93	2.59	3.609 (2)	134
C10—H10*A*⋯*Cg*2^ii^	0.93	2.91	3.788 (2)	156
C24—H24*A*⋯*Cg*3^iii^	0.93	2.96	3.676 (2)	136
C42—H42*A*⋯*Cg*4^iv^	0.93	2.81	3.641 (2)	155
C46—H46*C*⋯*Cg*5^v^	0.96	2.90	3.742 (2)	152

Symmetry codes: (i) 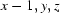; (ii) 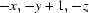; (iii) 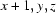; (iv) 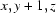; (v) 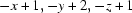.

**Fig. 1.**
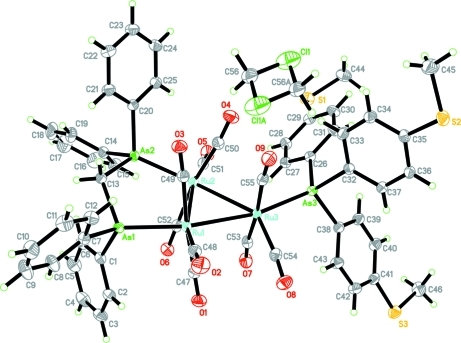
The molecular structure of the title compound with 50% probability ellipsoids for non-H atoms. Atoms with suffix A are generated by the symmetry operation (1 - x, 1 - y, -z).

**Fig. 2.**
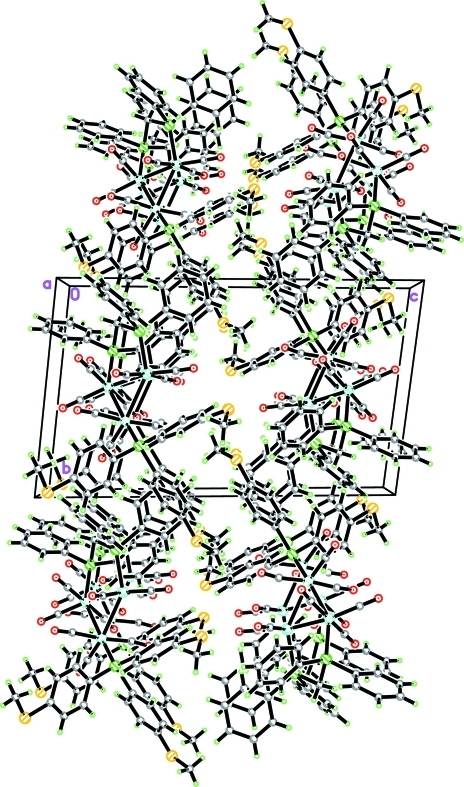
The crystal packing of the title compound, viewed down the a axis, showing the molecules stacking along the a axis. The solvent molecule have been omitted for clarity.

## supplementary crystallographic information

### Comment

*Triangulo*-triruthenium clusters are known for their interesting structural variations and related catalytic activity. A large number of substituted derivatives, Ru_3_(CO)_12-n_*L*_n_ (*L* = group 15 ligand) have been reported (Bruce *et al.*, 1985; Bruce, Liddell, Hughes *et al.*, 1988; Bruce, Liddell, Shawkataly *et al.*, 1988). As part of our study on the substitution of transition metal-carbonyl clusters with mixed-ligand complexes, we have published several structures of *triangulo*-triruthenium-carbonyl clusters containing mixed P/As and P/Sb ligands (Shawkataly *et al.*, 1998, 2004; Shawkataly, Khan, Sirat *et al.*, 2010*a*, *b*; Shawkataly, Khan, Yeap & Fun, 2010*a*, *b*). Herein we report the synthesis and structure of the title compound.

The asymmetry unit consists of one molecule of *triangulo*-triruthenium complex and half a molecule of dichloromethane solvent (Fig. 1). The dichloromethane solvent lies across a crystallographic inversion center leading to disorder of this solvent molecule over two positions. The geometric parameters of title compound are comparable to those found in related structures (Shawkataly, Khan, Sirat *et al.*, 2010*a*,*b*; Shawkataly, Khan, Yeap & Fun, 2010*a*, *b*). The bis(diphenylarsino)methane ligand bridges the Ru1—Ru2 bond and the monodentate arsine ligand bonds to the Ru3 atom. All arsine ligands are equatorial with respect to the Ru_3_ triangle. Additionally, each Ru atom carries one equatorial and two axial terminal carbonyl ligands. The three arsine-substituted benzene rings make dihedral angles (C26–C31/C32–C37, C26–C31/C38–C43 and C32–C37/C38–C43) of 70.02 (8), 82.85 (9) and 89.49 (8)° with each other respectively. The dihedral angles between the two benzene rings (C1–C6/C7–C12 and C14–C19/C20–C25) are 78.25 (9) and 86.59 (9)° for the two diphenylarsino groups respectively. The methylsulfanyl groups are nearly coplanar with the attached benzene rings [torsion angles C44–S1–C29–C30 = 10.75 (19), C45–S2–C35–C34 = -12.60 (18) and C46–S3–C41–C40 = 8.04 (18)°].

In the crystal packing, the molecules are stacked along *a* axis (Fig. 2). Weak intermolecular C—H···π interactions further stabilize the crystal structure (Table 1).

### Experimental

All manipulations were performed under a dry oxygen-free nitrogen atmosphere using standard Schlenk techniques. All solvents were dried over sodium and distilled from sodium benzophenone ketyl under dry oxygen-free nitrogen. Tris(4-(methylsulfanyl)phenyl)arsine was prepared from arsenic trichloride and 4-(methylsulfanyl)phenylmagnesium bromide in tetrahydrofuran and bis(diphenylarsino)methane (Bruce *et al.*, 1983) was prepared by reported procedure. The title compound was obtained by refluxing equimolar quantities of Ru_3_(CO)_10_(µ-Ph_2_AsCH_2_AsPh_2_) and tris(4-(methylsulfanyl)phenyl)arsine in hexane under nitrogen atmosphere. Crystals suitable for X-ray diffraction were grown by slow solvent / solvent diffusion of CH_3_OH into CH_2_Cl_2_.

### Refinement

All hydrogen atoms were positioned geometrically and refined using a riding model with C—H = 0.93–0.97 Å and *U*_iso_(H) = 1.2 or 1.5 *U*_eq_(C). Rotating group model was applied for the methyl groups. The highest peak and deepest hole in the difference Fourier map are located 0.74 and 0.65 Å, respectively, from atoms Ru1 and Cl1.

### Crystal data

**Table:**

2[Ru_3_(C_25_H_22_As_2_)(C_21_H_21_AsS_3_)(CO)_9_]·CH_2_Cl_2_	*Z* = 1
*M**_r_* = 3029.01	*F*(000) = 1490
Triclinic, *P*1	*D*_x_ = 1.810 Mg m^−^^3^
Hall symbol: -P 1	Mo *K*α radiation, λ = 0.71073 Å
*a* = 10.8807 (1) Å	Cell parameters from 9631 reflections
*b* = 12.7494 (2) Å	θ = 2.4–35.2°
*c* = 20.9320 (3) Å	µ = 2.79 mm^−^^1^
α = 94.512 (1)°	*T* = 100 K
β = 98.721 (1)°	Block, purple
γ = 102.855 (1)°	0.56 × 0.29 × 0.20 mm
*V* = 2779.13 (6) Å^3^	

### Data collection

**Table:**

Bruker SMART APEXII CCD area-detector diffractometer	20038 independent reflections
Radiation source: fine-focus sealed tube	17668 reflections with *I* > 2σ(*I*)
graphite	*R*_int_ = 0.027
φ and ω scans	θ_max_ = 32.5°, θ_min_ = 2.0°
Absorption correction: multi-scan (*SADABS*; Bruker, 2009)	*h* = −16→15
*T*_min_ = 0.306, *T*_max_ = 0.605	*k* = −19→19
117127 measured reflections	*l* = −31→31

### Refinement

**Table:**

Refinement on *F*^2^	Primary atom site location: structure-invariant direct methods
Least-squares matrix: full	Secondary atom site location: difference Fourier map
*R*[*F*^2^ > 2σ(*F*^2^)] = 0.023	Hydrogen site location: inferred from neighbouring sites
*wR*(*F*^2^) = 0.059	H-atom parameters constrained
*S* = 1.01	*w* = 1/[σ^2^(*F*_o_^2^) + (0.0279*P*)^2^ + 2.4001*P*] where *P* = (*F*_o_^2^ + 2*F*_c_^2^)/3
20038 reflections	(Δ/σ)_max_ = 0.002
679 parameters	Δρ_max_ = 1.53 e Å^−^^3^
0 restraints	Δρ_min_ = −1.28 e Å^−^^3^

### Special details

**Table:**

Experimental. The crystal was placed in the cold stream of an Oxford Cryosystems Cobra open-flow nitrogen cryostat (Cosier & Glazer, 1986) operating at 100.0 (1) K.
Geometry. All e.s.d.'s (except the e.s.d. in the dihedral angle between two l.s. planes) are estimated using the full covariance matrix. The cell e.s.d.'s are taken into account individually in the estimation of e.s.d.'s in distances, angles and torsion angles; correlations between e.s.d.'s in cell parameters are only used when they are defined by crystal symmetry. An approximate (isotropic) treatment of cell e.s.d.'s is used for estimating e.s.d.'s involving l.s. planes.
Refinement. Refinement of *F*^2^ against ALL reflections. The weighted *R*-factor *wR* and goodness of fit *S* are based on *F*^2^, conventional *R*-factors *R* are based on *F*, with *F* set to zero for negative *F*^2^. The threshold expression of *F*^2^ > σ(*F*^2^) is used only for calculating *R*-factors(gt) *etc*. and is not relevant to the choice of reflections for refinement. *R*-factors based on *F*^2^ are statistically about twice as large as those based on *F*, and *R*- factors based on ALL data will be even larger.

### Fractional atomic coordinates and isotropic or equivalent isotropic displacement parameters (Å^2^)

**Table:**

	*x*	*y*	*z*	*U*_iso_*/*U*_eq_	Occ. (<1)
Ru1	0.272408 (11)	0.645285 (10)	0.230778 (6)	0.01503 (3)	
Ru2	0.216711 (11)	0.432162 (10)	0.272952 (6)	0.01492 (3)	
Ru3	0.050554 (11)	0.491262 (10)	0.169550 (6)	0.01399 (3)	
As1	−0.111284 (14)	0.325798 (13)	0.168266 (7)	0.01466 (3)	
As2	0.125920 (15)	0.239793 (13)	0.239305 (8)	0.01539 (3)	
As3	0.488230 (15)	0.752248 (13)	0.279266 (8)	0.01564 (3)	
S1	0.85951 (5)	0.58930 (4)	0.50245 (2)	0.02778 (9)	
S2	0.82705 (5)	0.94466 (4)	0.06532 (2)	0.02522 (8)	
S3	0.46708 (5)	1.18489 (4)	0.45993 (2)	0.02596 (9)	
O1	0.37182 (14)	0.59457 (11)	0.10436 (7)	0.0281 (3)	
O2	0.18120 (13)	0.69186 (12)	0.35930 (7)	0.0282 (3)	
O3	0.14505 (14)	0.81485 (11)	0.17632 (7)	0.0293 (3)	
O4	0.38610 (13)	0.40882 (12)	0.39778 (7)	0.0297 (3)	
O5	0.43725 (13)	0.43147 (12)	0.19702 (7)	0.0293 (3)	
O6	0.01003 (12)	0.46497 (11)	0.35320 (6)	0.0241 (2)	
O7	0.19057 (13)	0.36294 (11)	0.08694 (6)	0.0256 (3)	
O8	−0.05033 (15)	0.58824 (13)	0.05006 (7)	0.0326 (3)	
O9	−0.07202 (13)	0.63293 (11)	0.25395 (7)	0.0277 (3)	
C1	−0.20996 (14)	0.31631 (13)	0.23781 (7)	0.0176 (3)	
C2	−0.27612 (16)	0.39622 (15)	0.24823 (8)	0.0211 (3)	
H2A	−0.2797	0.4475	0.2192	0.025*	
C3	−0.33693 (17)	0.39948 (17)	0.30216 (9)	0.0262 (3)	
H3A	−0.3807	0.4530	0.3091	0.031*	
C4	−0.33218 (18)	0.32294 (18)	0.34550 (9)	0.0298 (4)	
H4A	−0.3716	0.3258	0.3818	0.036*	
C5	−0.26847 (19)	0.24217 (18)	0.33449 (9)	0.0300 (4)	
H5A	−0.2669	0.1900	0.3630	0.036*	
C6	−0.20672 (16)	0.23860 (15)	0.28098 (8)	0.0225 (3)	
H6A	−0.1635	0.1846	0.2741	0.027*	
C7	−0.23627 (15)	0.27070 (13)	0.08947 (8)	0.0180 (3)	
C8	−0.36537 (17)	0.23264 (17)	0.08984 (9)	0.0264 (4)	
H8A	−0.3964	0.2347	0.1288	0.032*	
C9	−0.44931 (19)	0.19095 (19)	0.03128 (10)	0.0336 (4)	
H9A	−0.5363	0.1654	0.0314	0.040*	
C10	−0.4035 (2)	0.18778 (17)	−0.02634 (9)	0.0315 (4)	
H10A	−0.4595	0.1598	−0.0651	0.038*	
C11	−0.2739 (2)	0.22627 (17)	−0.02666 (9)	0.0286 (4)	
H11A	−0.2431	0.2239	−0.0657	0.034*	
C12	−0.19026 (17)	0.26813 (15)	0.03093 (8)	0.0232 (3)	
H12A	−0.1035	0.2945	0.0305	0.028*	
C13	−0.03139 (15)	0.20321 (13)	0.17414 (8)	0.0188 (3)	
H13A	−0.0115	0.1823	0.1320	0.023*	
H13B	−0.0910	0.1420	0.1857	0.023*	
C14	0.08324 (15)	0.14022 (14)	0.30329 (8)	0.0206 (3)	
C15	0.10313 (19)	0.17852 (16)	0.36886 (9)	0.0261 (3)	
H15A	0.1316	0.2524	0.3822	0.031*	
C16	0.0804 (2)	0.10586 (19)	0.41471 (11)	0.0369 (5)	
H16A	0.0942	0.1313	0.4587	0.044*	
C17	0.0370 (2)	−0.00437 (19)	0.39450 (12)	0.0414 (5)	
H17A	0.0211	−0.0526	0.4250	0.050*	
C18	0.0172 (2)	−0.04314 (18)	0.32887 (12)	0.0386 (5)	
H18A	−0.0116	−0.1171	0.3156	0.046*	
C19	0.04048 (19)	0.02893 (15)	0.28324 (10)	0.0282 (4)	
H19A	0.0277	0.0032	0.2393	0.034*	
C20	0.23331 (15)	0.16287 (13)	0.19779 (8)	0.0187 (3)	
C21	0.18807 (17)	0.09077 (15)	0.14123 (9)	0.0228 (3)	
H21A	0.1032	0.0790	0.1209	0.027*	
C22	0.27003 (18)	0.03630 (15)	0.11511 (9)	0.0256 (3)	
H22A	0.2403	−0.0111	0.0770	0.031*	
C23	0.39557 (18)	0.05293 (15)	0.14596 (10)	0.0267 (4)	
H23A	0.4502	0.0165	0.1285	0.032*	
C24	0.44057 (17)	0.12360 (15)	0.20272 (10)	0.0266 (4)	
H24A	0.5249	0.1338	0.2235	0.032*	
C25	0.35995 (16)	0.17917 (14)	0.22860 (9)	0.0222 (3)	
H25A	0.3904	0.2272	0.2664	0.027*	
C26	0.60497 (15)	0.69454 (13)	0.33824 (8)	0.0179 (3)	
C27	0.55549 (16)	0.63871 (14)	0.38685 (8)	0.0197 (3)	
H27A	0.4676	0.6218	0.3861	0.024*	
C28	0.63538 (17)	0.60794 (15)	0.43627 (8)	0.0219 (3)	
H28A	0.6010	0.5717	0.4687	0.026*	
C29	0.76761 (17)	0.63140 (14)	0.43739 (8)	0.0213 (3)	
C30	0.81776 (17)	0.68450 (16)	0.38786 (9)	0.0238 (3)	
H30A	0.9053	0.6987	0.3875	0.029*	
C31	0.73621 (16)	0.71633 (15)	0.33882 (8)	0.0222 (3)	
H31A	0.7701	0.7525	0.3062	0.027*	
C32	0.59651 (15)	0.80848 (13)	0.21796 (8)	0.0178 (3)	
C33	0.64666 (17)	0.73863 (14)	0.17964 (9)	0.0218 (3)	
H33A	0.6304	0.6653	0.1848	0.026*	
C34	0.72016 (17)	0.77792 (14)	0.13421 (9)	0.0223 (3)	
H34A	0.7547	0.7312	0.1099	0.027*	
C35	0.74257 (15)	0.88703 (14)	0.12467 (8)	0.0190 (3)	
C36	0.69174 (15)	0.95644 (13)	0.16237 (8)	0.0198 (3)	
H36A	0.7053	1.0292	0.1560	0.024*	
C37	0.62117 (15)	0.91777 (13)	0.20920 (8)	0.0188 (3)	
H37A	0.5902	0.9653	0.2349	0.023*	
C38	0.48767 (16)	0.88287 (13)	0.33407 (8)	0.0184 (3)	
C39	0.58630 (17)	0.92682 (14)	0.38610 (9)	0.0227 (3)	
H39A	0.6551	0.8948	0.3942	0.027*	
C40	0.58315 (18)	1.01828 (15)	0.42624 (9)	0.0241 (3)	
H40A	0.6491	1.0464	0.4612	0.029*	
C41	0.48100 (17)	1.06787 (14)	0.41394 (8)	0.0209 (3)	
C42	0.38201 (18)	1.02368 (15)	0.36202 (9)	0.0243 (3)	
H42A	0.3134	1.0559	0.3537	0.029*	
C43	0.38508 (17)	0.93201 (15)	0.32264 (9)	0.0231 (3)	
H43A	0.3181	0.9029	0.2882	0.028*	
C44	1.02155 (19)	0.65328 (19)	0.49658 (10)	0.0325 (4)	
H44A	1.0789	0.6379	0.5322	0.049*	
H44B	1.0310	0.7302	0.4981	0.049*	
H44C	1.0412	0.6259	0.4562	0.049*	
C45	0.9014 (2)	0.84077 (18)	0.03799 (11)	0.0369 (5)	
H45A	0.9614	0.8696	0.0109	0.055*	
H45B	0.8370	0.7813	0.0135	0.055*	
H45C	0.9454	0.8160	0.0749	0.055*	
C46	0.6196 (2)	1.22632 (18)	0.51238 (10)	0.0333 (4)	
H46A	0.6284	1.2970	0.5347	0.050*	
H46B	0.6864	1.2284	0.4870	0.050*	
H46C	0.6258	1.1757	0.5436	0.050*	
C47	0.33234 (16)	0.60875 (13)	0.15090 (8)	0.0202 (3)	
C48	0.21287 (16)	0.66581 (14)	0.31181 (8)	0.0211 (3)	
C49	0.20010 (16)	0.75351 (14)	0.19592 (8)	0.0211 (3)	
C50	0.32480 (16)	0.41959 (14)	0.35019 (8)	0.0211 (3)	
C51	0.35334 (16)	0.43797 (14)	0.22309 (8)	0.0210 (3)	
C52	0.08354 (16)	0.45379 (13)	0.32111 (8)	0.0194 (3)	
C53	0.14356 (15)	0.41231 (14)	0.11959 (8)	0.0193 (3)	
C54	−0.01435 (16)	0.55206 (14)	0.09543 (8)	0.0204 (3)	
C55	−0.02389 (15)	0.57839 (14)	0.22517 (8)	0.0194 (3)	
Cl1	0.61496 (8)	0.49986 (6)	0.04724 (4)	0.05599 (17)	
C56	0.4702 (4)	0.4354 (3)	0.0093 (2)	0.0350 (9)	0.50
H56A	0.4192	0.4153	0.0420	0.042*	0.50
H56B	0.4788	0.3697	−0.0133	0.042*	0.50

### Atomic displacement parameters (Å^2^)

**Table:**

	*U*^11^	*U*^22^	*U*^33^	*U*^12^	*U*^13^	*U*^23^
Ru1	0.01532 (5)	0.01408 (5)	0.01565 (5)	0.00296 (4)	0.00280 (4)	0.00285 (4)
Ru2	0.01539 (5)	0.01395 (5)	0.01464 (5)	0.00296 (4)	0.00039 (4)	0.00277 (4)
Ru3	0.01374 (5)	0.01517 (5)	0.01342 (5)	0.00404 (4)	0.00224 (4)	0.00237 (4)
As1	0.01356 (6)	0.01611 (7)	0.01415 (7)	0.00352 (5)	0.00181 (5)	0.00206 (5)
As2	0.01483 (6)	0.01406 (7)	0.01690 (7)	0.00340 (5)	0.00131 (5)	0.00233 (5)
As3	0.01571 (7)	0.01459 (7)	0.01686 (7)	0.00385 (5)	0.00312 (5)	0.00201 (5)
S1	0.0283 (2)	0.0360 (2)	0.02015 (19)	0.01163 (18)	−0.00015 (16)	0.00650 (17)
S2	0.0289 (2)	0.0235 (2)	0.0238 (2)	0.00305 (16)	0.01031 (16)	0.00445 (16)
S3	0.0324 (2)	0.0214 (2)	0.0243 (2)	0.00701 (17)	0.00700 (16)	−0.00196 (16)
O1	0.0348 (7)	0.0263 (7)	0.0255 (6)	0.0071 (5)	0.0118 (5)	0.0039 (5)
O2	0.0297 (7)	0.0293 (7)	0.0230 (6)	0.0003 (5)	0.0085 (5)	−0.0021 (5)
O3	0.0330 (7)	0.0267 (7)	0.0306 (7)	0.0124 (6)	0.0025 (5)	0.0078 (5)
O4	0.0287 (7)	0.0329 (7)	0.0247 (6)	0.0043 (5)	−0.0036 (5)	0.0104 (5)
O5	0.0242 (6)	0.0355 (7)	0.0346 (7)	0.0131 (5)	0.0104 (5)	0.0148 (6)
O6	0.0237 (6)	0.0285 (6)	0.0201 (6)	0.0054 (5)	0.0044 (4)	0.0034 (5)
O7	0.0255 (6)	0.0286 (7)	0.0234 (6)	0.0089 (5)	0.0051 (5)	−0.0027 (5)
O8	0.0372 (8)	0.0368 (8)	0.0266 (7)	0.0141 (6)	0.0015 (6)	0.0129 (6)
O9	0.0254 (6)	0.0293 (7)	0.0303 (7)	0.0098 (5)	0.0081 (5)	−0.0019 (5)
C1	0.0148 (6)	0.0216 (7)	0.0156 (6)	0.0025 (5)	0.0023 (5)	0.0025 (5)
C2	0.0186 (7)	0.0252 (8)	0.0200 (7)	0.0055 (6)	0.0045 (5)	0.0033 (6)
C3	0.0202 (7)	0.0358 (10)	0.0229 (8)	0.0077 (7)	0.0057 (6)	−0.0012 (7)
C4	0.0231 (8)	0.0457 (12)	0.0202 (8)	0.0035 (8)	0.0078 (6)	0.0061 (8)
C5	0.0275 (9)	0.0385 (11)	0.0250 (8)	0.0045 (8)	0.0073 (7)	0.0138 (8)
C6	0.0198 (7)	0.0252 (8)	0.0226 (8)	0.0041 (6)	0.0040 (6)	0.0074 (6)
C7	0.0182 (6)	0.0186 (7)	0.0163 (6)	0.0040 (5)	0.0005 (5)	0.0026 (5)
C8	0.0201 (7)	0.0362 (10)	0.0197 (7)	0.0008 (7)	0.0008 (6)	0.0052 (7)
C9	0.0226 (8)	0.0427 (12)	0.0280 (9)	−0.0029 (8)	−0.0038 (7)	0.0055 (8)
C10	0.0329 (9)	0.0332 (10)	0.0225 (8)	0.0050 (8)	−0.0076 (7)	−0.0007 (7)
C11	0.0357 (10)	0.0338 (10)	0.0166 (7)	0.0121 (8)	0.0011 (7)	0.0004 (7)
C12	0.0242 (8)	0.0278 (9)	0.0183 (7)	0.0082 (6)	0.0033 (6)	0.0021 (6)
C13	0.0164 (6)	0.0172 (7)	0.0218 (7)	0.0047 (5)	0.0001 (5)	0.0006 (6)
C14	0.0179 (7)	0.0202 (7)	0.0244 (8)	0.0043 (6)	0.0037 (6)	0.0076 (6)
C15	0.0319 (9)	0.0251 (9)	0.0238 (8)	0.0081 (7)	0.0090 (7)	0.0069 (7)
C16	0.0492 (12)	0.0389 (11)	0.0288 (10)	0.0136 (10)	0.0163 (9)	0.0146 (9)
C17	0.0501 (13)	0.0350 (11)	0.0430 (12)	0.0064 (10)	0.0160 (10)	0.0228 (10)
C18	0.0409 (11)	0.0230 (9)	0.0498 (13)	−0.0013 (8)	0.0095 (10)	0.0140 (9)
C19	0.0298 (9)	0.0205 (8)	0.0314 (9)	0.0004 (7)	0.0037 (7)	0.0059 (7)
C20	0.0177 (6)	0.0167 (7)	0.0225 (7)	0.0051 (5)	0.0037 (5)	0.0041 (6)
C21	0.0222 (7)	0.0226 (8)	0.0239 (8)	0.0083 (6)	0.0018 (6)	0.0002 (6)
C22	0.0297 (9)	0.0242 (8)	0.0253 (8)	0.0118 (7)	0.0049 (7)	0.0009 (7)
C23	0.0255 (8)	0.0244 (8)	0.0345 (9)	0.0111 (7)	0.0100 (7)	0.0046 (7)
C24	0.0180 (7)	0.0256 (9)	0.0374 (10)	0.0081 (6)	0.0039 (7)	0.0048 (7)
C25	0.0182 (7)	0.0201 (8)	0.0272 (8)	0.0050 (6)	0.0006 (6)	0.0016 (6)
C26	0.0189 (7)	0.0181 (7)	0.0169 (7)	0.0054 (5)	0.0023 (5)	0.0016 (5)
C27	0.0194 (7)	0.0194 (7)	0.0201 (7)	0.0042 (6)	0.0036 (5)	0.0018 (6)
C28	0.0228 (7)	0.0240 (8)	0.0189 (7)	0.0050 (6)	0.0036 (6)	0.0040 (6)
C29	0.0244 (7)	0.0219 (8)	0.0176 (7)	0.0079 (6)	0.0012 (6)	0.0010 (6)
C30	0.0199 (7)	0.0309 (9)	0.0227 (8)	0.0097 (6)	0.0036 (6)	0.0058 (7)
C31	0.0198 (7)	0.0266 (8)	0.0224 (8)	0.0077 (6)	0.0052 (6)	0.0076 (6)
C32	0.0172 (6)	0.0179 (7)	0.0182 (7)	0.0037 (5)	0.0028 (5)	0.0027 (5)
C33	0.0260 (8)	0.0162 (7)	0.0241 (8)	0.0045 (6)	0.0079 (6)	0.0029 (6)
C34	0.0253 (8)	0.0197 (8)	0.0227 (8)	0.0057 (6)	0.0077 (6)	0.0001 (6)
C35	0.0179 (7)	0.0198 (7)	0.0174 (7)	0.0015 (5)	0.0022 (5)	0.0017 (6)
C36	0.0189 (7)	0.0163 (7)	0.0237 (7)	0.0030 (5)	0.0031 (6)	0.0036 (6)
C37	0.0185 (7)	0.0169 (7)	0.0207 (7)	0.0041 (5)	0.0035 (5)	0.0011 (6)
C38	0.0203 (7)	0.0164 (7)	0.0188 (7)	0.0046 (5)	0.0043 (5)	0.0019 (5)
C39	0.0222 (7)	0.0207 (8)	0.0242 (8)	0.0056 (6)	0.0009 (6)	0.0014 (6)
C40	0.0271 (8)	0.0209 (8)	0.0223 (8)	0.0057 (6)	−0.0001 (6)	−0.0004 (6)
C41	0.0272 (8)	0.0170 (7)	0.0198 (7)	0.0054 (6)	0.0082 (6)	0.0025 (6)
C42	0.0252 (8)	0.0237 (8)	0.0252 (8)	0.0096 (6)	0.0041 (6)	0.0005 (6)
C43	0.0235 (7)	0.0230 (8)	0.0229 (8)	0.0080 (6)	0.0014 (6)	−0.0002 (6)
C44	0.0257 (9)	0.0432 (12)	0.0301 (9)	0.0143 (8)	0.0003 (7)	0.0045 (8)
C45	0.0490 (13)	0.0332 (11)	0.0345 (11)	0.0128 (9)	0.0222 (9)	0.0024 (8)
C46	0.0355 (10)	0.0325 (10)	0.0286 (9)	0.0035 (8)	0.0077 (8)	−0.0062 (8)
C47	0.0223 (7)	0.0161 (7)	0.0217 (7)	0.0036 (6)	0.0031 (6)	0.0037 (6)
C48	0.0190 (7)	0.0209 (8)	0.0213 (7)	0.0009 (6)	0.0023 (6)	0.0032 (6)
C49	0.0231 (7)	0.0204 (7)	0.0194 (7)	0.0036 (6)	0.0045 (6)	0.0031 (6)
C50	0.0203 (7)	0.0198 (7)	0.0221 (7)	0.0027 (6)	0.0018 (6)	0.0044 (6)
C51	0.0198 (7)	0.0211 (8)	0.0219 (7)	0.0051 (6)	0.0007 (6)	0.0076 (6)
C52	0.0207 (7)	0.0183 (7)	0.0167 (7)	0.0025 (6)	−0.0013 (5)	0.0026 (5)
C53	0.0188 (7)	0.0196 (7)	0.0186 (7)	0.0035 (6)	0.0019 (5)	0.0027 (6)
C54	0.0197 (7)	0.0222 (8)	0.0198 (7)	0.0059 (6)	0.0035 (5)	0.0034 (6)
C55	0.0191 (7)	0.0209 (7)	0.0187 (7)	0.0054 (6)	0.0034 (5)	0.0026 (6)
Cl1	0.0662 (4)	0.0542 (4)	0.0519 (4)	0.0291 (3)	0.0076 (3)	−0.0050 (3)
C56	0.040 (2)	0.0269 (19)	0.042 (2)	0.0097 (16)	0.0186 (18)	0.0030 (17)

### Geometric parameters (Å, °)

**Table:**

Ru1—C49	1.8768 (17)	C15—C16	1.397 (3)
Ru1—C48	1.9246 (17)	C15—H15A	0.9300
Ru1—C47	1.9445 (17)	C16—C17	1.388 (3)
Ru1—As3	2.4574 (2)	C16—H16A	0.9300
Ru1—Ru3	2.80754 (17)	C17—C18	1.391 (4)
Ru1—Ru2	2.88731 (18)	C17—H17A	0.9300
Ru2—C50	1.8897 (17)	C18—C19	1.389 (3)
Ru2—C51	1.9335 (18)	C18—H18A	0.9300
Ru2—C52	1.9414 (17)	C19—H19A	0.9300
Ru2—As2	2.4344 (2)	C20—C21	1.393 (2)
Ru2—Ru3	2.86304 (17)	C20—C25	1.393 (2)
Ru3—C54	1.8993 (17)	C21—C22	1.393 (2)
Ru3—C55	1.9309 (17)	C21—H21A	0.9300
Ru3—C53	1.9338 (17)	C22—C23	1.382 (3)
Ru3—As1	2.4240 (2)	C22—H22A	0.9300
As1—C1	1.9314 (16)	C23—C24	1.386 (3)
As1—C7	1.9431 (16)	C23—H23A	0.9300
As1—C13	1.9534 (16)	C24—C25	1.388 (3)
As2—C20	1.9406 (16)	C24—H24A	0.9300
As2—C14	1.9532 (16)	C25—H25A	0.9300
As2—C13	1.9608 (16)	C26—C31	1.391 (2)
As3—C26	1.9395 (16)	C26—C27	1.393 (2)
As3—C32	1.9432 (16)	C27—C28	1.387 (2)
As3—C38	1.9482 (16)	C27—H27A	0.9300
S1—C29	1.7560 (17)	C28—C29	1.399 (2)
S1—C44	1.797 (2)	C28—H28A	0.9300
S2—C35	1.7657 (17)	C29—C30	1.396 (2)
S2—C45	1.798 (2)	C30—C31	1.398 (2)
S3—C41	1.7589 (17)	C30—H30A	0.9300
S3—C46	1.790 (2)	C31—H31A	0.9300
O1—C47	1.140 (2)	C32—C37	1.391 (2)
O2—C48	1.149 (2)	C32—C33	1.404 (2)
O3—C49	1.150 (2)	C33—C34	1.387 (2)
O4—C50	1.148 (2)	C33—H33A	0.9300
O5—C51	1.147 (2)	C34—C35	1.394 (2)
O6—C52	1.144 (2)	C34—H34A	0.9300
O7—C53	1.143 (2)	C35—C36	1.397 (2)
O8—C54	1.142 (2)	C36—C37	1.389 (2)
O9—C55	1.146 (2)	C36—H36A	0.9300
C1—C2	1.394 (2)	C37—H37A	0.9300
C1—C6	1.395 (2)	C38—C39	1.391 (2)
C2—C3	1.395 (2)	C38—C43	1.396 (2)
C2—H2A	0.9300	C39—C40	1.392 (3)
C3—C4	1.388 (3)	C39—H39A	0.9300
C3—H3A	0.9300	C40—C41	1.397 (2)
C4—C5	1.387 (3)	C40—H40A	0.9300
C4—H4A	0.9300	C41—C42	1.392 (3)
C5—C6	1.394 (3)	C42—C43	1.386 (3)
C5—H5A	0.9300	C42—H42A	0.9300
C6—H6A	0.9300	C43—H43A	0.9300
C7—C8	1.382 (2)	C44—H44A	0.9600
C7—C12	1.393 (2)	C44—H44B	0.9600
C8—C9	1.400 (3)	C44—H44C	0.9600
C8—H8A	0.9300	C45—H45A	0.9600
C9—C10	1.374 (3)	C45—H45B	0.9600
C9—H9A	0.9300	C45—H45C	0.9600
C10—C11	1.388 (3)	C46—H46A	0.9600
C10—H10A	0.9300	C46—H46B	0.9600
C11—C12	1.384 (2)	C46—H46C	0.9600
C11—H11A	0.9300	Cl1—C56	1.652 (5)
C12—H12A	0.9300	Cl1—C56^i^	1.761 (5)
C13—H13A	0.9700	C56—Cl1^i^	1.761 (5)
C13—H13B	0.9700	C56—H56A	0.9599
C14—C15	1.388 (3)	C56—H56B	0.9599
C14—C19	1.400 (3)		
			
C49—Ru1—C48	92.55 (7)	C16—C17—H17A	119.8
C49—Ru1—C47	92.99 (7)	C18—C17—H17A	119.8
C48—Ru1—C47	173.85 (7)	C19—C18—C17	119.8 (2)
C49—Ru1—As3	100.49 (5)	C19—C18—H18A	120.1
C48—Ru1—As3	91.09 (5)	C17—C18—H18A	120.1
C47—Ru1—As3	90.53 (5)	C18—C19—C14	120.0 (2)
C49—Ru1—Ru3	88.60 (5)	C18—C19—H19A	120.0
C48—Ru1—Ru3	95.13 (5)	C14—C19—H19A	120.0
C47—Ru1—Ru3	82.34 (5)	C21—C20—C25	119.90 (16)
As3—Ru1—Ru3	168.757 (7)	C21—C20—As2	123.14 (12)
C49—Ru1—Ru2	144.64 (5)	C25—C20—As2	116.92 (13)
C48—Ru1—Ru2	75.31 (5)	C20—C21—C22	119.93 (16)
C47—Ru1—Ru2	98.60 (5)	C20—C21—H21A	120.0
As3—Ru1—Ru2	112.611 (6)	C22—C21—H21A	120.0
Ru3—Ru1—Ru2	60.344 (4)	C23—C22—C21	119.83 (18)
C50—Ru2—C51	91.86 (7)	C23—C22—H22A	120.1
C50—Ru2—C52	91.39 (7)	C21—C22—H22A	120.1
C51—Ru2—C52	169.68 (7)	C22—C23—C24	120.43 (17)
C50—Ru2—As2	97.87 (5)	C22—C23—H23A	119.8
C51—Ru2—As2	93.44 (5)	C24—C23—H23A	119.8
C52—Ru2—As2	95.82 (5)	C23—C24—C25	120.09 (17)
C50—Ru2—Ru3	168.52 (5)	C23—C24—H24A	120.0
C51—Ru2—Ru3	93.27 (5)	C25—C24—H24A	120.0
C52—Ru2—Ru3	81.86 (5)	C24—C25—C20	119.82 (17)
As2—Ru2—Ru3	92.069 (6)	C24—C25—H25A	120.1
C50—Ru2—Ru1	113.64 (5)	C20—C25—H25A	120.1
C51—Ru2—Ru1	72.94 (5)	C31—C26—C27	118.87 (15)
C52—Ru2—Ru1	96.79 (5)	C31—C26—As3	123.38 (12)
As2—Ru2—Ru1	145.613 (7)	C27—C26—As3	117.35 (12)
Ru3—Ru2—Ru1	58.448 (5)	C28—C27—C26	120.97 (15)
C54—Ru3—C55	90.94 (7)	C28—C27—H27A	119.5
C54—Ru3—C53	92.15 (7)	C26—C27—H27A	119.5
C55—Ru3—C53	173.56 (7)	C27—C28—C29	120.10 (16)
C54—Ru3—As1	103.19 (5)	C27—C28—H28A	120.0
C55—Ru3—As1	94.78 (5)	C29—C28—H28A	120.0
C53—Ru3—As1	90.01 (5)	C30—C29—C28	119.37 (15)
C54—Ru3—Ru1	105.36 (5)	C30—C29—S1	124.46 (13)
C55—Ru3—Ru1	80.28 (5)	C28—C29—S1	116.17 (13)
C53—Ru3—Ru1	93.45 (5)	C29—C30—C31	119.87 (16)
As1—Ru3—Ru1	151.077 (7)	C29—C30—H30A	120.1
C54—Ru3—Ru2	163.58 (5)	C31—C30—H30A	120.1
C55—Ru3—Ru2	95.58 (5)	C26—C31—C30	120.78 (16)
C53—Ru3—Ru2	79.99 (5)	C26—C31—H31A	119.6
As1—Ru3—Ru2	91.288 (6)	C30—C31—H31A	119.6
Ru1—Ru3—Ru2	61.208 (4)	C37—C32—C33	118.79 (15)
C1—As1—C7	104.88 (7)	C37—C32—As3	120.65 (12)
C1—As1—C13	103.91 (7)	C33—C32—As3	120.51 (12)
C7—As1—C13	98.75 (7)	C34—C33—C32	120.63 (16)
C1—As1—Ru3	117.77 (5)	C34—C33—H33A	119.7
C7—As1—Ru3	119.04 (5)	C32—C33—H33A	119.7
C13—As1—Ru3	109.90 (5)	C33—C34—C35	120.42 (16)
C20—As2—C14	97.80 (7)	C33—C34—H34A	119.8
C20—As2—C13	101.07 (7)	C35—C34—H34A	119.8
C14—As2—C13	102.17 (7)	C34—C35—C36	118.99 (15)
C20—As2—Ru2	115.82 (5)	C34—C35—S2	124.43 (13)
C14—As2—Ru2	120.82 (5)	C36—C35—S2	116.54 (13)
C13—As2—Ru2	115.87 (5)	C37—C36—C35	120.63 (15)
C26—As3—C32	101.35 (7)	C37—C36—H36A	119.7
C26—As3—C38	99.20 (7)	C35—C36—H36A	119.7
C32—As3—C38	102.56 (7)	C36—C37—C32	120.51 (15)
C26—As3—Ru1	121.96 (5)	C36—C37—H37A	119.7
C32—As3—Ru1	115.64 (5)	C32—C37—H37A	119.7
C38—As3—Ru1	113.24 (5)	C39—C38—C43	118.88 (16)
C29—S1—C44	103.72 (9)	C39—C38—As3	121.12 (12)
C35—S2—C45	103.76 (9)	C43—C38—As3	119.97 (13)
C41—S3—C46	102.37 (9)	C38—C39—C40	120.73 (16)
C2—C1—C6	119.66 (15)	C38—C39—H39A	119.6
C2—C1—As1	118.17 (12)	C40—C39—H39A	119.6
C6—C1—As1	121.88 (13)	C39—C40—C41	120.05 (16)
C1—C2—C3	120.11 (16)	C39—C40—H40A	120.0
C1—C2—H2A	119.9	C41—C40—H40A	120.0
C3—C2—H2A	119.9	C42—C41—C40	119.24 (16)
C4—C3—C2	120.12 (18)	C42—C41—S3	116.25 (13)
C4—C3—H3A	119.9	C40—C41—S3	124.51 (14)
C2—C3—H3A	119.9	C43—C42—C41	120.43 (16)
C5—C4—C3	119.80 (17)	C43—C42—H42A	119.8
C5—C4—H4A	120.1	C41—C42—H42A	119.8
C3—C4—H4A	120.1	C42—C43—C38	120.67 (16)
C4—C5—C6	120.52 (17)	C42—C43—H43A	119.7
C4—C5—H5A	119.7	C38—C43—H43A	119.7
C6—C5—H5A	119.7	S1—C44—H44A	109.5
C5—C6—C1	119.76 (17)	S1—C44—H44B	109.5
C5—C6—H6A	120.1	H44A—C44—H44B	109.5
C1—C6—H6A	120.1	S1—C44—H44C	109.5
C8—C7—C12	119.96 (15)	H44A—C44—H44C	109.5
C8—C7—As1	122.94 (12)	H44B—C44—H44C	109.5
C12—C7—As1	117.08 (12)	S2—C45—H45A	109.5
C7—C8—C9	119.75 (17)	S2—C45—H45B	109.5
C7—C8—H8A	120.1	H45A—C45—H45B	109.5
C9—C8—H8A	120.1	S2—C45—H45C	109.5
C10—C9—C8	120.16 (18)	H45A—C45—H45C	109.5
C10—C9—H9A	119.9	H45B—C45—H45C	109.5
C8—C9—H9A	119.9	S3—C46—H46A	109.5
C9—C10—C11	120.02 (17)	S3—C46—H46B	109.5
C9—C10—H10A	120.0	H46A—C46—H46B	109.5
C11—C10—H10A	120.0	S3—C46—H46C	109.5
C12—C11—C10	120.23 (18)	H46A—C46—H46C	109.5
C12—C11—H11A	119.9	H46B—C46—H46C	109.5
C10—C11—H11A	119.9	O1—C47—Ru1	175.32 (15)
C11—C12—C7	119.86 (17)	O2—C48—Ru1	171.28 (15)
C11—C12—H12A	120.1	O3—C49—Ru1	173.42 (16)
C7—C12—H12A	120.1	O4—C50—Ru2	177.06 (16)
As1—C13—As2	111.17 (8)	O5—C51—Ru2	172.10 (15)
As1—C13—H13A	109.4	O6—C52—Ru2	175.31 (14)
As2—C13—H13A	109.4	O7—C53—Ru3	175.10 (15)
As1—C13—H13B	109.4	O8—C54—Ru3	177.98 (16)
As2—C13—H13B	109.4	O9—C55—Ru3	174.75 (15)
H13A—C13—H13B	108.0	C56—Cl1—C56^i^	60.8 (3)
C15—C14—C19	119.98 (16)	Cl1—C56—C56^i^	62.8 (3)
C15—C14—As2	120.30 (13)	Cl1—C56—Cl1^i^	119.2 (2)
C19—C14—As2	119.58 (14)	C56^i^—C56—Cl1^i^	56.5 (3)
C14—C15—C16	119.89 (19)	Cl1—C56—H56A	107.5
C14—C15—H15A	120.1	C56^i^—C56—H56A	127.4
C16—C15—H15A	120.1	Cl1^i^—C56—H56A	108.3
C17—C16—C15	119.9 (2)	Cl1—C56—H56B	106.7
C17—C16—H16A	120.1	C56^i^—C56—H56B	125.3
C15—C16—H16A	120.1	Cl1^i^—C56—H56B	107.3
C16—C17—C18	120.42 (19)	H56A—C56—H56B	107.3
			
C49—Ru1—Ru2—C50	−138.92 (11)	C7—As1—C1—C2	−79.09 (14)
C48—Ru1—Ru2—C50	−65.79 (8)	C13—As1—C1—C2	177.73 (13)
C47—Ru1—Ru2—C50	113.36 (8)	Ru3—As1—C1—C2	55.95 (14)
As3—Ru1—Ru2—C50	19.17 (6)	C7—As1—C1—C6	107.05 (14)
Ru3—Ru1—Ru2—C50	−170.60 (6)	C13—As1—C1—C6	3.88 (15)
C49—Ru1—Ru2—C51	136.67 (10)	Ru3—As1—C1—C6	−117.91 (13)
C48—Ru1—Ru2—C51	−150.20 (7)	C6—C1—C2—C3	1.1 (2)
C47—Ru1—Ru2—C51	28.95 (7)	As1—C1—C2—C3	−172.93 (13)
As3—Ru1—Ru2—C51	−65.25 (5)	C1—C2—C3—C4	−0.2 (3)
Ru3—Ru1—Ru2—C51	104.98 (5)	C2—C3—C4—C5	−1.0 (3)
C49—Ru1—Ru2—C52	−44.40 (10)	C3—C4—C5—C6	1.4 (3)
C48—Ru1—Ru2—C52	28.73 (7)	C4—C5—C6—C1	−0.6 (3)
C47—Ru1—Ru2—C52	−152.12 (7)	C2—C1—C6—C5	−0.7 (3)
As3—Ru1—Ru2—C52	113.69 (5)	As1—C1—C6—C5	173.10 (14)
Ru3—Ru1—Ru2—C52	−76.08 (5)	C1—As1—C7—C8	−1.40 (17)
C49—Ru1—Ru2—As2	66.38 (9)	C13—As1—C7—C8	105.61 (16)
C48—Ru1—Ru2—As2	139.51 (5)	Ru3—As1—C7—C8	−135.74 (14)
C47—Ru1—Ru2—As2	−41.34 (5)	C1—As1—C7—C12	−179.85 (13)
As3—Ru1—Ru2—As2	−135.534 (11)	C13—As1—C7—C12	−72.84 (14)
Ru3—Ru1—Ru2—As2	34.694 (11)	Ru3—As1—C7—C12	45.81 (15)
C49—Ru1—Ru2—Ru3	31.68 (9)	C12—C7—C8—C9	0.4 (3)
C48—Ru1—Ru2—Ru3	104.81 (5)	As1—C7—C8—C9	−177.99 (16)
C47—Ru1—Ru2—Ru3	−76.04 (5)	C7—C8—C9—C10	0.1 (3)
As3—Ru1—Ru2—Ru3	−170.228 (7)	C8—C9—C10—C11	−0.2 (3)
C49—Ru1—Ru3—C54	27.93 (7)	C9—C10—C11—C12	−0.1 (3)
C48—Ru1—Ru3—C54	120.36 (7)	C10—C11—C12—C7	0.6 (3)
C47—Ru1—Ru3—C54	−65.28 (7)	C8—C7—C12—C11	−0.7 (3)
As3—Ru1—Ru3—C54	−116.29 (6)	As1—C7—C12—C11	177.76 (14)
Ru2—Ru1—Ru3—C54	−169.77 (5)	C1—As1—C13—As2	−83.61 (9)
C49—Ru1—Ru3—C55	−60.37 (7)	C7—As1—C13—As2	168.59 (8)
C48—Ru1—Ru3—C55	32.06 (7)	Ru3—As1—C13—As2	43.27 (9)
C47—Ru1—Ru3—C55	−153.57 (7)	C20—As2—C13—As1	−142.99 (8)
As3—Ru1—Ru3—C55	155.41 (6)	C14—As2—C13—As1	116.42 (9)
Ru2—Ru1—Ru3—C55	101.93 (5)	Ru2—As2—C13—As1	−16.97 (10)
C49—Ru1—Ru3—C53	121.11 (7)	C20—As2—C14—C15	127.08 (15)
C48—Ru1—Ru3—C53	−146.46 (7)	C13—As2—C14—C15	−129.76 (15)
C47—Ru1—Ru3—C53	27.90 (7)	Ru2—As2—C14—C15	0.66 (16)
As3—Ru1—Ru3—C53	−23.11 (6)	C20—As2—C14—C19	−48.66 (15)
Ru2—Ru1—Ru3—C53	−76.59 (5)	C13—As2—C14—C19	54.50 (15)
C49—Ru1—Ru3—As1	−142.62 (5)	Ru2—As2—C14—C19	−175.08 (12)
C48—Ru1—Ru3—As1	−50.18 (5)	C19—C14—C15—C16	0.0 (3)
C47—Ru1—Ru3—As1	124.18 (5)	As2—C14—C15—C16	−175.76 (16)
As3—Ru1—Ru3—As1	73.16 (4)	C14—C15—C16—C17	−0.5 (3)
Ru2—Ru1—Ru3—As1	19.685 (13)	C15—C16—C17—C18	0.6 (4)
C49—Ru1—Ru3—Ru2	−162.30 (5)	C16—C17—C18—C19	−0.2 (4)
C48—Ru1—Ru3—Ru2	−69.87 (5)	C17—C18—C19—C14	−0.3 (3)
C47—Ru1—Ru3—Ru2	104.50 (5)	C15—C14—C19—C18	0.4 (3)
As3—Ru1—Ru3—Ru2	53.48 (3)	As2—C14—C19—C18	176.16 (16)
C50—Ru2—Ru3—C54	86.0 (3)	C14—As2—C20—C21	95.77 (15)
C51—Ru2—Ru3—C54	−30.4 (2)	C13—As2—C20—C21	−8.33 (16)
C52—Ru2—Ru3—C54	140.46 (19)	Ru2—As2—C20—C21	−134.38 (13)
As2—Ru2—Ru3—C54	−123.96 (19)	C14—As2—C20—C25	−81.72 (14)
Ru1—Ru2—Ru3—C54	37.28 (19)	C13—As2—C20—C25	174.18 (13)
C50—Ru2—Ru3—C55	−27.0 (3)	Ru2—As2—C20—C25	48.13 (14)
C51—Ru2—Ru3—C55	−143.36 (7)	C25—C20—C21—C22	−0.9 (3)
C52—Ru2—Ru3—C55	27.50 (7)	As2—C20—C21—C22	−178.32 (14)
As2—Ru2—Ru3—C55	123.08 (5)	C20—C21—C22—C23	0.9 (3)
Ru1—Ru2—Ru3—C55	−75.69 (5)	C21—C22—C23—C24	−0.1 (3)
C50—Ru2—Ru3—C53	148.3 (3)	C22—C23—C24—C25	−0.7 (3)
C51—Ru2—Ru3—C53	31.93 (7)	C23—C24—C25—C20	0.7 (3)
C52—Ru2—Ru3—C53	−157.22 (7)	C21—C20—C25—C24	0.1 (3)
As2—Ru2—Ru3—C53	−61.64 (5)	As2—C20—C25—C24	177.69 (14)
Ru1—Ru2—Ru3—C53	99.60 (5)	C32—As3—C26—C31	12.40 (16)
C50—Ru2—Ru3—As1	−121.9 (3)	C38—As3—C26—C31	−92.50 (15)
C51—Ru2—Ru3—As1	121.71 (5)	Ru1—As3—C26—C31	142.60 (13)
C52—Ru2—Ru3—As1	−67.44 (5)	C32—As3—C26—C27	−174.97 (13)
As2—Ru2—Ru3—As1	28.143 (7)	C38—As3—C26—C27	80.12 (14)
Ru1—Ru2—Ru3—As1	−170.622 (7)	Ru1—As3—C26—C27	−44.78 (15)
C50—Ru2—Ru3—Ru1	48.7 (3)	C31—C26—C27—C28	1.9 (3)
C51—Ru2—Ru3—Ru1	−67.67 (5)	As3—C26—C27—C28	−171.04 (13)
C52—Ru2—Ru3—Ru1	103.18 (5)	C26—C27—C28—C29	−0.9 (3)
As2—Ru2—Ru3—Ru1	−161.235 (7)	C27—C28—C29—C30	−0.9 (3)
C54—Ru3—As1—C1	−113.07 (7)	C27—C28—C29—S1	179.66 (14)
C55—Ru3—As1—C1	−20.97 (7)	C44—S1—C29—C30	10.75 (19)
C53—Ru3—As1—C1	154.72 (7)	C44—S1—C29—C28	−169.88 (15)
Ru1—Ru3—As1—C1	57.57 (6)	C28—C29—C30—C31	1.8 (3)
Ru2—Ru3—As1—C1	74.74 (5)	S1—C29—C30—C31	−178.86 (14)
C54—Ru3—As1—C7	15.56 (8)	C27—C26—C31—C30	−1.1 (3)
C55—Ru3—As1—C7	107.66 (7)	As3—C26—C31—C30	171.47 (14)
C53—Ru3—As1—C7	−76.65 (7)	C29—C30—C31—C26	−0.8 (3)
Ru1—Ru3—As1—C7	−173.80 (6)	C26—As3—C32—C37	−120.37 (13)
Ru2—Ru3—As1—C7	−156.63 (6)	C38—As3—C32—C37	−18.14 (15)
C54—Ru3—As1—C13	128.27 (7)	Ru1—As3—C32—C37	105.59 (13)
C55—Ru3—As1—C13	−139.63 (7)	C26—As3—C32—C33	62.45 (14)
C53—Ru3—As1—C13	36.06 (7)	C38—As3—C32—C33	164.68 (13)
Ru1—Ru3—As1—C13	−61.09 (5)	Ru1—As3—C32—C33	−71.59 (14)
Ru2—Ru3—As1—C13	−43.92 (5)	C37—C32—C33—C34	0.4 (3)
C50—Ru2—As2—C20	−78.97 (8)	As3—C32—C33—C34	177.68 (13)
C51—Ru2—As2—C20	13.39 (7)	C32—C33—C34—C35	−1.7 (3)
C52—Ru2—As2—C20	−171.19 (7)	C33—C34—C35—C36	1.1 (3)
Ru3—Ru2—As2—C20	106.79 (5)	C33—C34—C35—S2	−176.40 (14)
Ru1—Ru2—As2—C20	77.75 (5)	C45—S2—C35—C34	−12.60 (18)
C50—Ru2—As2—C14	38.70 (8)	C45—S2—C35—C36	169.84 (14)
C51—Ru2—As2—C14	131.05 (7)	C34—C35—C36—C37	0.7 (2)
C52—Ru2—As2—C14	−53.52 (7)	S2—C35—C36—C37	178.44 (13)
Ru3—Ru2—As2—C14	−135.55 (6)	C35—C36—C37—C32	−2.0 (2)
Ru1—Ru2—As2—C14	−164.58 (6)	C33—C32—C37—C36	1.4 (2)
C50—Ru2—As2—C13	162.89 (8)	As3—C32—C37—C36	−175.82 (12)
C51—Ru2—As2—C13	−104.76 (7)	C26—As3—C38—C39	20.61 (15)
C52—Ru2—As2—C13	70.67 (7)	C32—As3—C38—C39	−83.30 (15)
Ru3—Ru2—As2—C13	−11.35 (6)	Ru1—As3—C38—C39	151.38 (12)
Ru1—Ru2—As2—C13	−40.39 (6)	C26—As3—C38—C43	−157.37 (14)
C49—Ru1—As3—C26	168.10 (8)	C32—As3—C38—C43	98.72 (14)
C48—Ru1—As3—C26	75.30 (8)	Ru1—As3—C38—C43	−26.59 (15)
C47—Ru1—As3—C26	−98.77 (7)	C43—C38—C39—C40	0.0 (3)
Ru3—Ru1—As3—C26	−48.38 (7)	As3—C38—C39—C40	−178.00 (14)
Ru2—Ru1—As3—C26	0.78 (6)	C38—C39—C40—C41	−0.9 (3)
C49—Ru1—As3—C32	−68.08 (8)	C39—C40—C41—C42	1.1 (3)
C48—Ru1—As3—C32	−160.87 (7)	C39—C40—C41—S3	−178.79 (14)
C47—Ru1—As3—C32	25.06 (7)	C46—S3—C41—C42	−171.86 (15)
Ru3—Ru1—As3—C32	75.45 (6)	C46—S3—C41—C40	8.04 (18)
Ru2—Ru1—As3—C32	124.61 (5)	C40—C41—C42—C43	−0.5 (3)
C49—Ru1—As3—C38	49.87 (7)	S3—C41—C42—C43	179.43 (14)
C48—Ru1—As3—C38	−42.92 (7)	C41—C42—C43—C38	−0.4 (3)
C47—Ru1—As3—C38	143.01 (7)	C39—C38—C43—C42	0.6 (3)
Ru3—Ru1—As3—C38	−166.60 (6)	As3—C38—C43—C42	178.66 (14)
Ru2—Ru1—As3—C38	−117.44 (5)		

Symmetry codes: (i) −*x*+1, −*y*+1, −*z*.

### Hydrogen-bond geometry (Å, °)

**Table:**

Cg1, Cg2, Cg3, Cg4 and Cg5 are the centroids of the C26–C31, C32–C37, C1–C6, C14–C19 and C38–C43 benzene rings, respectively.

**Table:**

*D*—H···*A*	*D*—H	H···*A*	*D*···*A*	*D*—H···*A*
C3—H3A···Cg1^ii^	0.93	2.59	3.609 (2)	134
C10—H10A···Cg2^iii^	0.93	2.91	3.788 (2)	156
C24—H24A···Cg3^iv^	0.93	2.96	3.676 (2)	136
C42—H42A···Cg4^v^	0.93	2.81	3.641 (2)	155
C46—H46C···Cg5^vi^	0.96	2.90	3.742 (2)	152

Symmetry codes: (ii) *x*−1, *y*, *z*; (iii) −*x*, −*y*+1, −*z*; (iv) *x*+1, *y*, *z*; (v) *x*, *y*+1, *z*; (vi) −*x*+1, −*y*+2, −*z*+1.
